# The venous system of E14.5 mouse embryos—reference data and examples for diagnosing malformations in embryos with gene deletions

**DOI:** 10.1111/joa.13536

**Published:** 2021-08-25

**Authors:** Stefan H. Geyer, Barbara Maurer‐Gesek, Lukas F. Reissig, Julia Rose, Fabrice Prin, Robert Wilson, Antonella Galli, Catherine Tudor, Jacqueline K. White, Timothy J. Mohun, Wolfgang J. Weninger

**Affiliations:** ^1^ Division of Anatomy MIC BioImaging Austria/CMI Medical University of Vienna Vienna Austria; ^2^ Crick Advanced Light Microscopy Facility The Francis Crick Institute London UK; ^3^ The Francis Crick Institute London UK; ^4^ Wellcome Trust Sanger Institute Wellcome Genome Campus Cambridge UK

**Keywords:** DMDD, embryo, HREM, mouse, mutant, phenotyping, venous system

## Abstract

Approximately one‐third of randomly produced knockout mouse lines produce homozygous offspring, which fail to survive the perinatal period. The majority of these die around or after embryonic day (E)14.5, presumably from cardiovascular insufficiency. For diagnosing structural abnormalities underlying death and diseases and for researching gene function, the phenotype of these individuals has to be analysed. This makes the creation of reference data, which define normal anatomy and normal variations the highest priority. While such data do exist for the heart and arteries, they are still missing for the venous system. Here we provide high‐quality descriptive and metric information on the normal anatomy of the venous system of E14.5 embryos. Using high‐resolution digital volume data and 3D models from 206 genetically normal embryos, bred on the C57BL/6N background, we present precise descriptive and metric information of the venous system as it presents itself in each of the six developmental stages of E14.5. The resulting data shed new light on the maturation and remodelling of the venous system at transition of embryo to foetal life and provide a reference that can be used for detecting venous abnormalities in mutants. To explore this capacity, we analysed the venous phenotype of embryos from 7 knockout lines (*Atp11a*, *Morc2a*, *1700067K01Rik*, *B9d2*, *Oaz1*, *Celf4* and *Coro1c*). Careful comparisons enabled the diagnosis of not only simple malformations, such as dual inferior vena cava, but also complex and subtle abnormalities, which would have escaped diagnosis in the absence of detailed, stage‐specific referenced data.

## Graphical abstract

We provide high‐quality descriptive and metric information on the normal anatomy of the venous system of E14.5 mouse embryos. Our data shed new light on the maturation and remodelling of the venous system at the transition of embryo to foetal life and provide a reference that can be used for detecting venous abnormalities in mutants. Using our data, we were able to diagnose not only simple malformations but also complex and subtle abnormalities of embryos of seven knockout lines.

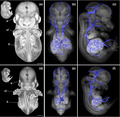

## 1 INTRODUCTION

About 2% of human newborns worldwide are diagnosed as being affected by a hereditary disease (Dolk et al., 2010; EUROCAT, 2018; Morris et al., 2018). In many cases, this has negative effects on health and life quality and often results in a dramatically reduced life span. The suffering of the affected individuals, together with the highly negative socioeconomic impact, emphasises the urgency of developing novel diagnostic and therapeutic strategies. The basis for such a development is profound knowledge of the effects genetic, biomechanical and environmental factors have on normal embryo‐ and foetogenesis and peri‐ and postnatal development.

Luckily, gene function and basic developmental mechanisms are evolutionary conserved and a number of species, including drosophila, zebrafish, xenopus and the chick can be employed as models for investigating the triggers and driving factors of basal events in embryogenesis, tissue formation and remodelling. The most important model however is the mouse (Brown et al., 2018; Rosenthal & Brown, 2007). It is a species that is relatively closely related to humans, having a largely similar body plan. Furthermore, with a short reproduction time, a variety of tools for manipulating the mouse genome have been established. By analysing the phenotype of altered individuals, the effect of genetic, epigenetic or biomechanical factors on the formation and remodelling of tissue and organs can be explored. To facilitate the identification of phenotypic abnormalities, detailed descriptions of the normal anatomy and metric data of essential organ systems at important stages of intra‐ and extrauterine development have been made available (Captur et al., 2016; Desgrange et al., 2019; Geyer et al., 2017c; Weninger et al., 2009; Wong et al., 2014). Using them as a reference, they permit the diagnosis of phenotype abnormalities in genetically engineered or compromised individuals.

Systematic knockout efforts, initiated by the international mouse phenotyping consortium (IMPC, www.mousephenotype.org), demonstrated that a third of the mouse genes are essential for embryo development and growth, since full deletion resulted in pre‐ or perinatal death of homozygous individuals (Ayadi et al., 2012; Dickinson et al., 2016). In these lines, careful phenotyping of embryos is the only possibility to diagnose the full spectrum of organ and tissue abnormalities linked to the function of the deleted gene. As identified in the program ‘Deciphering the Mechanisms of Developmental Disorders’ (DMDD), embryonic day (E)14.5 is the most important time point for such phenotype analyses (Mohun et al., 2013; Weninger et al., 2014, Wilson et al., 2016b). This is for several reasons: First, organogenesis is largely finished at E14.5, thereby permitting the detection of effects gene deletions have on the formation of all major organ systems relevant for embryo survival, growth and development; second, roughly half of the embryos of peri‐ or prenatally lethal mutant mouse lines survive until E14.5; third, a large proportion of embryos compromised by severe cardiovascular or endocrine malfunctions is still alive or at least not fully resorbed after intrauterine death and therefore still available for phenotype analysis (Geyer et al., 2017b; Mohun et al., 2013).

Another lesson learned from DMDD is that gene deletions in E14.5 embryos quite often result in subtle structural organ or tissue defects. The identification of such defects is challenging and requires the employment of cutting‐edge three‐dimensional (3D) imaging techniques. Since the high‐resolution episcopic imaging (HREM) technique (Mohun & Weninger, 2011; Weninger et al., 2006) routinely provides digital volume data with voxel dimensions down to 3 × 3 × 3 µm^3^ from whole E14.5 mouse embryos, DMDD and an increasing number of stand‐alone projects employ this imaging method for phenotyping (Geyer & Weninger, 2019; Weninger et al., 2018). Its basis are series of two‐dimensional (2D) digital images which nearly match the quality of images derived from glass slide mounted histological sections. However, HREM images are captured from subsequently exposed surfaces of a resin block during its sectioning on a microtome. Therefore, the single images do not show artefacts introduced by section processing and the full series of such images are precisely aligned, facilitating their simple conversion to a high‐quality volume data.

Traditionally, phenotyping relies on direct comparisons of mutants with genetically normal littermates, harvested at the same time point and, ideally, stemming from the same dam. Recently, it was demonstrated that this approach is highly error prone, because developmental progress, size, morphology and topology of mouse embryos and their organs and tissues harvested at E14.5 can vary dramatically. Comparing littermates without acknowledging stage differences and at the same time neglecting the spectrum of natural variation in the normal population is prone to cause false diagnosis of malformations and abnormalities. To overcome this problem, a staging system specifically for embryos harvested at E14.5 has been developed. It distinguishes 6 stages (S21, S22^−^, S22, S22^+^, S23^−^ and S23) of developmental progress, each showing several unique features, which might be considered as abnormal at other stages (Geyer et al., 2017b).

Using this staging system, careful descriptions of the morphology and topology of the arterial system of E14.5 mouse embryos have recently been generated for each of the six identified stages. These data have been successfully used as reference for diagnosing cardiovascular malformations in experimentally challenged and genetically engineered embryos (Geyer et al., 2017c). Although of similar importance for a comprehensive analysis of the cardiovascular system, comparable reference data defining the normal morphology of the venous system of E14.5 do not currently exist. We therefore decided to use careful 3D descriptions, analyses and visualisations of the venous anatomy of genetically normal E14.5 mouse embryos to provide such reference data, identifying the normal anatomical features of the venous system and exploring normal variations across the six successive stages of E14.5 development.

## 2 MATERIALS AND METHODS

The phenotypes of 206 wild‐type mouse embryos of the C57BL/6N strain and 56 mutants of 7 different knockout lines (*Atp11a*, *Morc2a*, *1700067K01Rik*, *B9d2*, *Oaz1*, *Celf4* and *Coro1c*) were comprehensively analysed (Table 1). All embryos were generated at the Wellcome Trust Sanger Institute (http://www.sanger.ac.uk) as part of the DMDD project (https://dmdd.org.uk) and its pilot, with embryos harvested at embryonic day (E) 14.5 and 13.5, respectively. The precise developmental stage of each embryo was subsequently determined using criteria based upon forelimb maturation (Geyer et al., 2017b).

**TABLE 1:** Numbers of wild‐type embryos analysed in this study

Stage	Number
21	31
22−	15
22	30
22+	47
23−	38
23	45
Total	206

Of the wild‐type mouse embryos, 175 were harvested at E14.5 and 31 were harvested at E13.5. The latter were selected embryos of late E13.5 stages, which overlap in appearance with early E14.5 substages. This increased the number of S21 embryos—a stage, which holds late E13.5, in addition to a small proportion of early E14.5 embryos.

For all 206 embryos, 3D computer representations were created using high‐resolution episcopic microscopy (Weninger et al., 2006). As given in standard protocols, the embryos were fixed in Bouin's fixative for 24 h (minimum) and washed in phosphate‐buffered saline (Geyer et al., 2017a; Mohun & Weninger, 2012a, 2012b). They were then dehydrated in methanol (70%, 80%, 90%, 95%, 100%, 2 h each) before infiltration and embedding in JB4 resin (JB4, Polysciences). Eosin (0.275 g/100 ml) and acridine orange (0.055 g/100 ml) were added to the infiltration and embedding solutions. Blocks were sealed air‐tight and allowed to polymerise at room temperature for at least 24 h, before they were baked at 90℃ for 1–2 days.

**FIGURE 1:**
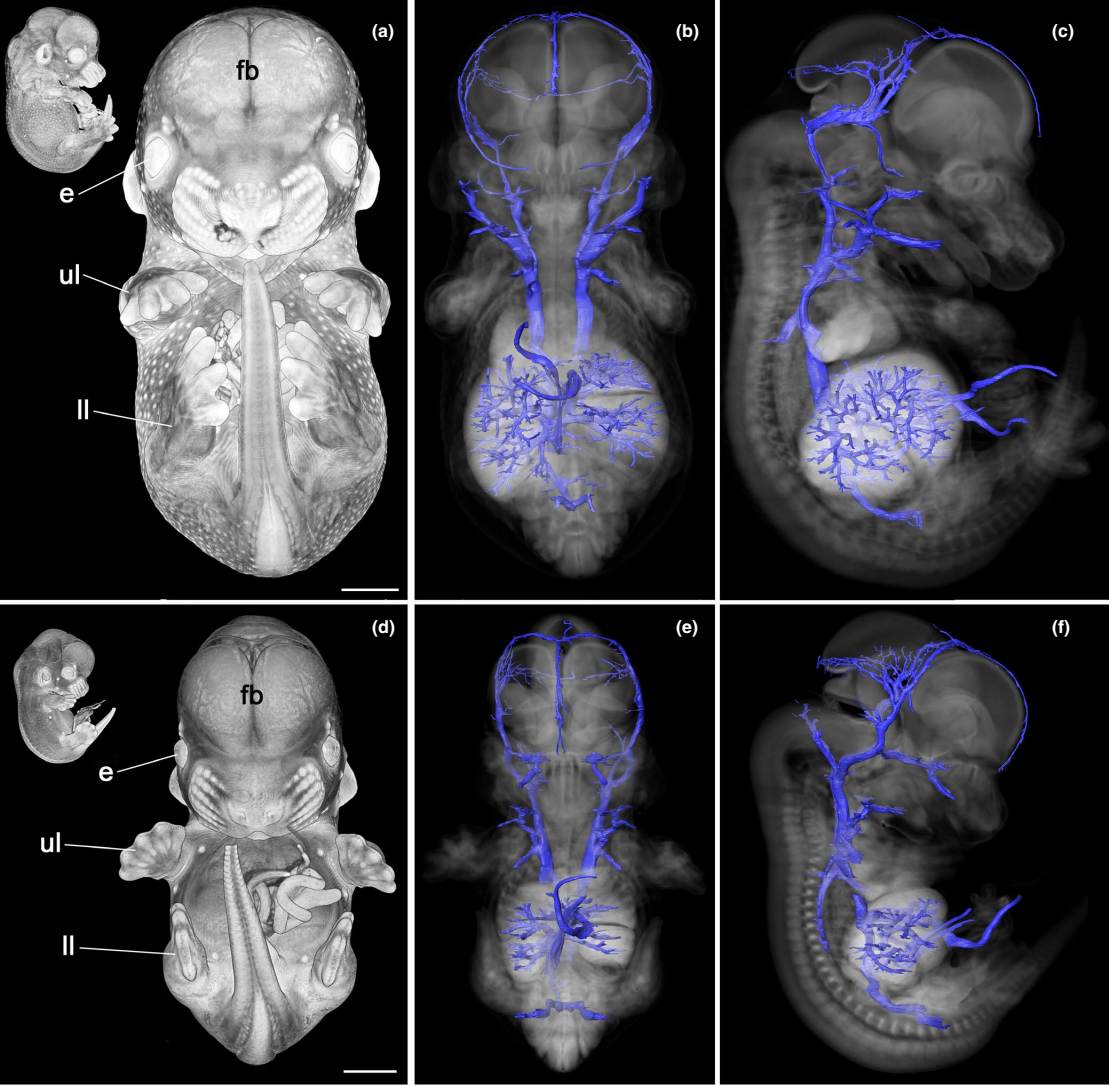
Venous system of E14.5 mouse embryos. Developmental stage 23 (a–c) and stage 21 (d–f). Volume‐rendered 3D model of the embryo from ventral and from the right (inlay) (a, d). 3D surface‐rendered models of the veins (blue) in context of a semi‐transparent volume model of the embryo from ventral (b, e) and right (c, f). e, eye; fb, forebrain; ll, lower limb; ul, upper limb. Scale bars 1 mm [Colour figure can be viewed at wileyonlinelibrary.com]

Utilising a commercially available Optical‐HREM system (Indigo Scientific Limited), 3000–4000 single digital images (pixel dimension 1–2 µm) of subsequently exposed block faces were generated. Each series comprised a whole embryo. Since section thickness, and thus the distance between subsequent block face images ranged between 2.5 and 3.5 µm, the two‐dimensional images were downscaled to gain isotropic voxel dimensions, before being virtually stacked. The virtual image stacks were converted to volume datasets and visualised employing Osirix Software (OsiriX v5.6, 64bit, Pixmeo Sarl), which ran on a MacPro Computer (3 GHz‐8‐Core Intel Xeon E5, 64 GB RAM, AMD FirePro D700). Using the straight and oblique section and 3D volume rendering tools, the data were carefully analysed following a detailed protocol for screening morphologic phenotypes of E14.5 embryos (Weninger et al., 2014). In 12 wild‐type mouse embryos (Stages (S)21, 22−, 22, 22+, 23− and 23), the venous system was manually segmented and surface‐rendered models were generated using the Amira software (versions 5.4.5 and 6.4.0, Thermo Fisher Scientific, Merignac, France; Figure 1), which was operated on PC workstations, equipped with 192 GB RAM and a NVIDIA Titan XP graphic card.

Statistical analyses were performed using Excel (Microsoft Office 2013 for Windows) and SPSS (IBM SPSS Statistics Version 22). Stage dependency in the presence of the sigmoid and primary head sinus was tested using Fisher's exact test, as contingency tables included cells with numbers <5. *p* values <0.05 were considered statistically significant.

## 3 RESULTS

**FIGURE 2:**
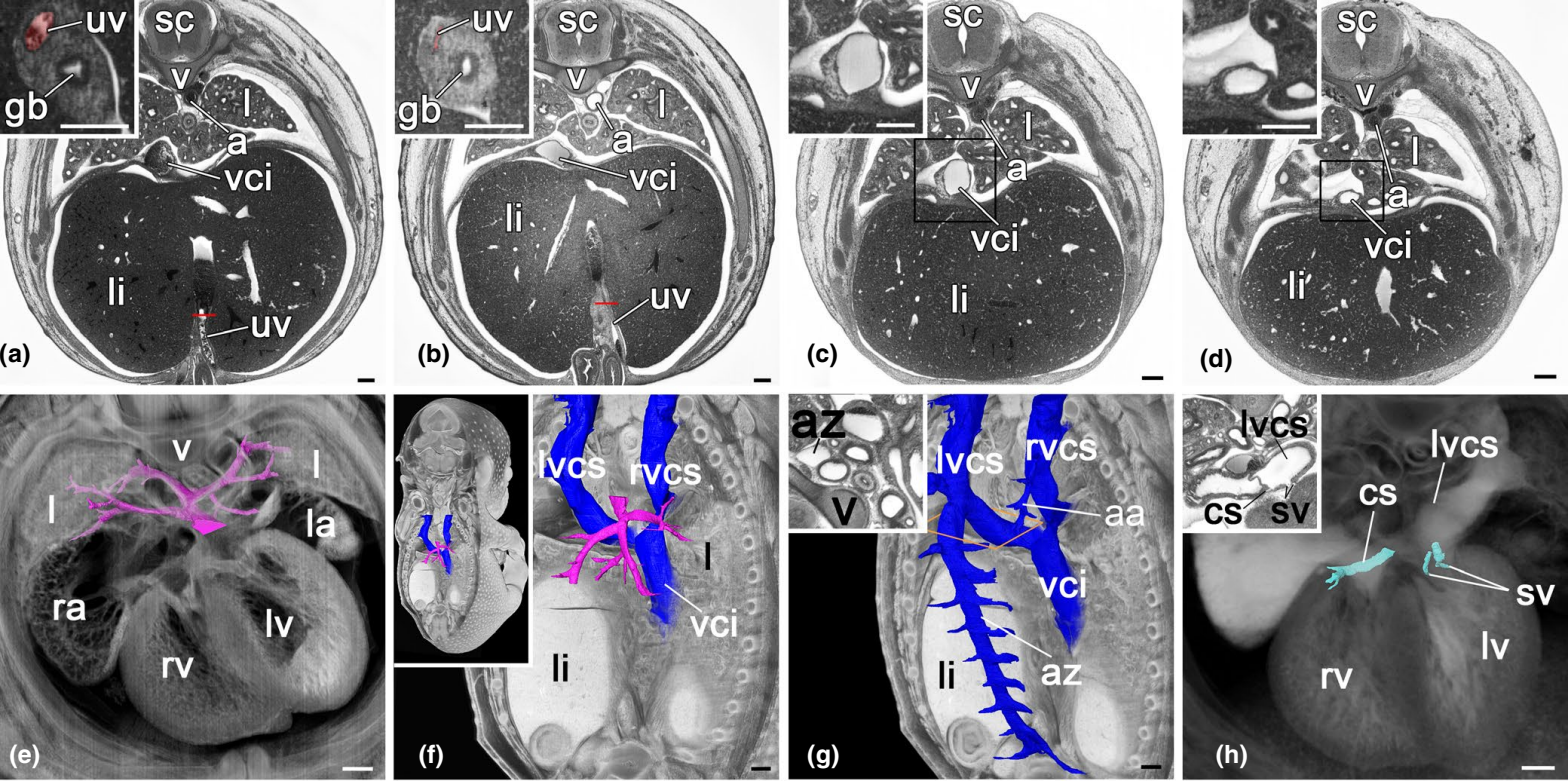
Veins of E14.5 mouse embryos. (a–d) Axial ‘High‐resolution episcopic microscopy’ (HREM)‐sections, view from cranial. Top of the images is dorsal. (a, b) Variable appearance of the umbilical vein (uv). Inlays display virtual coronal re‐sections through region marked in (a) and (b). Note the compressed, slit‐like umbilical vein in (b). (c, d) Variable dimensions of inferior vena cava (vci). (e–g) Pulmonary veins, venae cavae and vena azygos. (e) Surface model of the pulmonary veins (magenta) in context of a volume‐rendered model sectioned axially, seen from cranial. (f, g) Surface model of pulmonary veins (magenta) (f), venae cavae (blue) (f, g) and vena azygos (blue) (g) in context with frontally sectioned volume model seen from right back. Axial HREM section (inlay) through region marked in (g). (h) Cardiac veins. Surface model of veins in context of semi‐transparent volume model sectioned axially, seen from cranial. Top of the image is dorsal. a, aorta; aa, accessory azygos vein; az, azygos vein; cs, coronary sinus; gb, gallbladder; l, lung; la, left atrium; li, liver; lv, left ventricle; lvcs, left vena cava superior; ra, right atrium; rv, right ventricle; rvcs, right vena cava superior; sc, spinal cord; sv, single veins; uv, umbilical vein; v, vertebra; vci; vena cava inferior. Scale bars 200 µm [Colour figure can be viewed at wileyonlinelibrary.com]

In general, veins do appear in HREM data as irregularly shaped or thin, slit‐like structures. This is especially true for the umbilical and internal jugular vein and the inferior vena cava. In addition, the dimensions of corresponding veins differ significantly between individuals, even between individuals of identical E14.5 stages (S; Figure 2).

### 3.1 Pulmonary veins

In all embryos, a single lung vein enters the left atrium from the dorsal side. It crosses over the left superior vena cava and opens into the left atrium immediately left to the septum primum. The single vein receives one vessel from the left and two from the right lung. The latter drain the right cranial, middle, caudal and accessory lobes (Figure 2).

### 3.2 Systemic veins

Three venae cavae enter the right atrium. One inferior vena cava enters from the caudal side. It starts with the connection of the two common iliac veins, ascends in the retroperitoneum, penetrates the diaphragm and runs for a relatively long distance inside the thorax, before it enters the heart.

A right superior vena cava enters the heart straight from cranial side. A left superior vena cava enters from left lateral side, after undercrossing the single lung vein. Both venae cavae sup. are formed at the level of the upper thoracic aperture from the confluence of the ipsilateral subclavian and internal and external jugular veins. The left receives the azygous vein and the right a short vein stem from its caudal side. Both receive a variable number of small vein stems cranially, which collect blood from the three cranial intercostal spaces (Figure 2).

#### 3.2.1 Cardiac veins

While undercrossing the single pulmonary vein, the left superior vena cava receives the coronary sinus, as well as 2–3 single veins of variable dimensions coming directly from the myocardium of the left ventricle (Figure 2).

#### 3.2.2 Intracranial venous system

**FIGURE 3:**
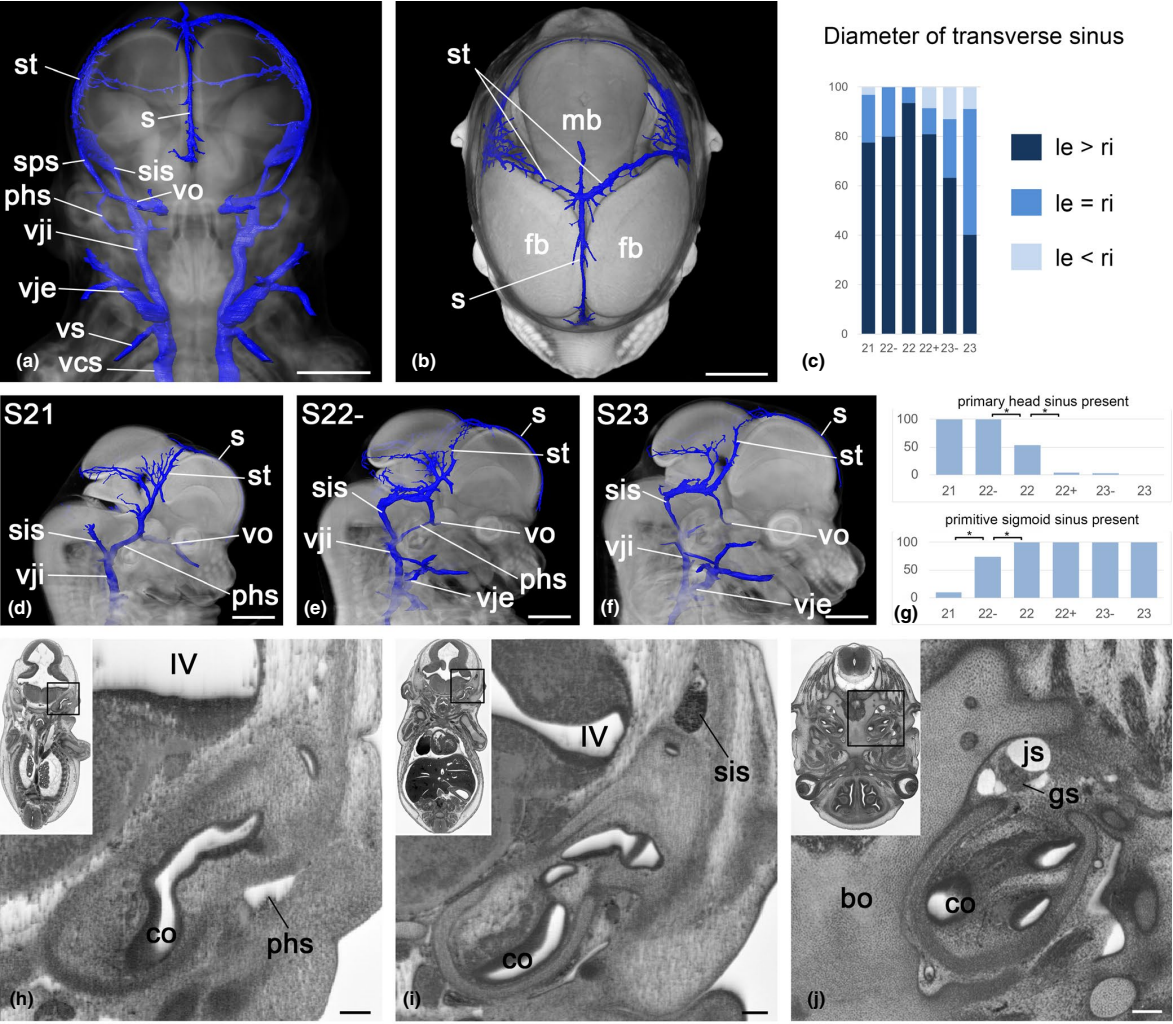
Head veins. (a, b) Surface models of veins and volume models of the head. View from ventral (a) and view from top (b). Note the larger diameter of the right transverse sinus (st) (b). (c) Percentage of embryos with differing dimensions of left and right transverse sinus, *n* = 206. (d–f) Surface model of head veins in context with volume model of head seen from right. Note the changes of the primary head sinus (phs) and the sigmoid sinus (sis) from stages 21 to 23. (g) Percentage of embryos with present primary head sinus and sigmoid sinus, respectively, *n* = 206. *p < 0.05 with Fisher's exact test. (h, i) Coronal re‐section through HREM data. Note the position of the primary head sinus (phs) (h) and primitive sigmoid sinus (sis) (i). (j) Jugular foramen. Axial section from cranial. bo, basisphenoid bone; co, cochlea; fb, forebrain; gs, superior ganglion of the glossopharyngeal nerve; IV, fourth ventricle; js, junction of sigmoid sinus and jugular vein; mb, midbrain; phs, primary head sinus; s, sagittal sinus; sis, sigmoid sinus; st, transverse sinus; vcs; vena cava superior; vje, external jugular vein; vji, internal jugular vein; vo, ophthalmic vein; vs, subclavian vein. Scale bars 1 mm (a, b, d–f), 100 µm (h–j) [Colour figure can be viewed at wileyonlinelibrary.com]

All embryos show a forming superior sagittal and transverse sinus. The primitive superior sagittal sinus extends in the midline from anterior to the transition of fore‐ and midbrain. It receives several veins from both telencephalic hemispheres. The primitive transverse sinus extends bilaterally and receives veins from the fore‐ and midbrain. In the majority of embryos younger than S23^−^, the diameter on the left sinus is distinguishably larger than the right. At S23, about 50% have equal dimensions (Figure 3).

All embryos of S22 and later exhibit a continuous channel, which we identified as the primitive sigmoid sinus. This channel is only present in 10% of S21 embryos and only three‐quarters (73%) of those at S22^−^. Instead of a continuous channel, these earlier embryos display unconnected extensions of the transverse sinus and jugular vein to various lengths. In addition to the extensions, the younger embryos have a very prominent venous channel of large diameter, which connects the ophthalmic vein in the region of the later cavernous sinus with the jugular vein. We identified this channel as the primary head sinus. It runs ventrolaterally to the auditory vesicle and joins the internal jugular vein in the neck. In S22 embryos, the diameter of this channel varies heavily. In S23 embryos, it does not exist any longer. Applying Fisher's exact test revealed significant differences regarding the presence of the primary head sinus at the transition from S22^−^ to S22 (*p* = 0.001) and from S22 to S22+ (*p *< 0.001). Similarly, differences were also detected between the S21 and S22^−^ (*p* < 0.001) and stages 22^−^ and 22 (*p* = 0.009) for the presence of a continuous sigmoid sinus (Figure 3).

All embryos show an ophthalmic vein starting in the orbit. It passes laterally to the pituitary gland and in early embryos drains into the primary head sinus. In later stages, the vessel appears to connect directly to the primitive sigmoid sinus, having a constant diameter with no visible enlargements or narrowings in the region of the later cavernous or superior petrosal sinus.

#### 3.2.3 Internal and external jugular and subclavian veins

The internal jugular vein starts at the jugular foramen, which is a large gap between cochlea and occipital bone in early embryos and an almost osseous canal in later embryos (Figure 3).

It descends in the neck, ventral to the sympathetic trunk and in close proximity to the carotid artery and vagus nerve. At the level of the superior thoracic aperture, it is joined by the external jugular vein from the ventral side and the subclavian vein from lateral side. Before they join, the subclavian vein passes through the well‐developed anterior scalenus gap.

#### 3.2.4 Azygos vein

The azygos vein starts in the left‐sided retroperitoneum, where it is joined by numerous small local veins. Many of them form anastomoses with vessels draining into the inferior vena cava. The main stem of the vessel ascends left to the aorta showing a steadily increasing diameter. It then penetrates the diaphragm, receives veins from the left‐sided caudal intercostal spaces and from cranial up to three channels, which drain the first 3 left‐sided intercostal spaces. Finally, it drains into the left superior vena cava at approximately the level of the fourth thoracic vertebra.

On the right side, a short accessory azygos vein drains caudally into the right superior vena cava. It starts inside the thoracic cavity by the confluence of the caudal, right‐sided intercostal veins and receives up to three venous channels from cranial side.

#### 3.2.5 Inferior vena cava

The inferior vena cava starts at the junction of the common iliac veins and runs in the retroperitoneum right to the abdominal aorta. It receives local vessels and the renal veins at the level of the second lumbar vertebra. The left renal vein crosses over the aorta and passes straight through the ganglion material of the paraaortic bodies.

Immediately after receiving the renal veins, the inferior vena cava bends anteriorly, enters the liver tissue and ascends in a rightwards directed curve towards the diaphragm. Independent of precise stage, the liver segment of the vessel receives the ductus venosus, which starts between the lateral liver lobes and connects to the inferior vena cava inside the right medial liver lobe.

**FIGURE 4:**
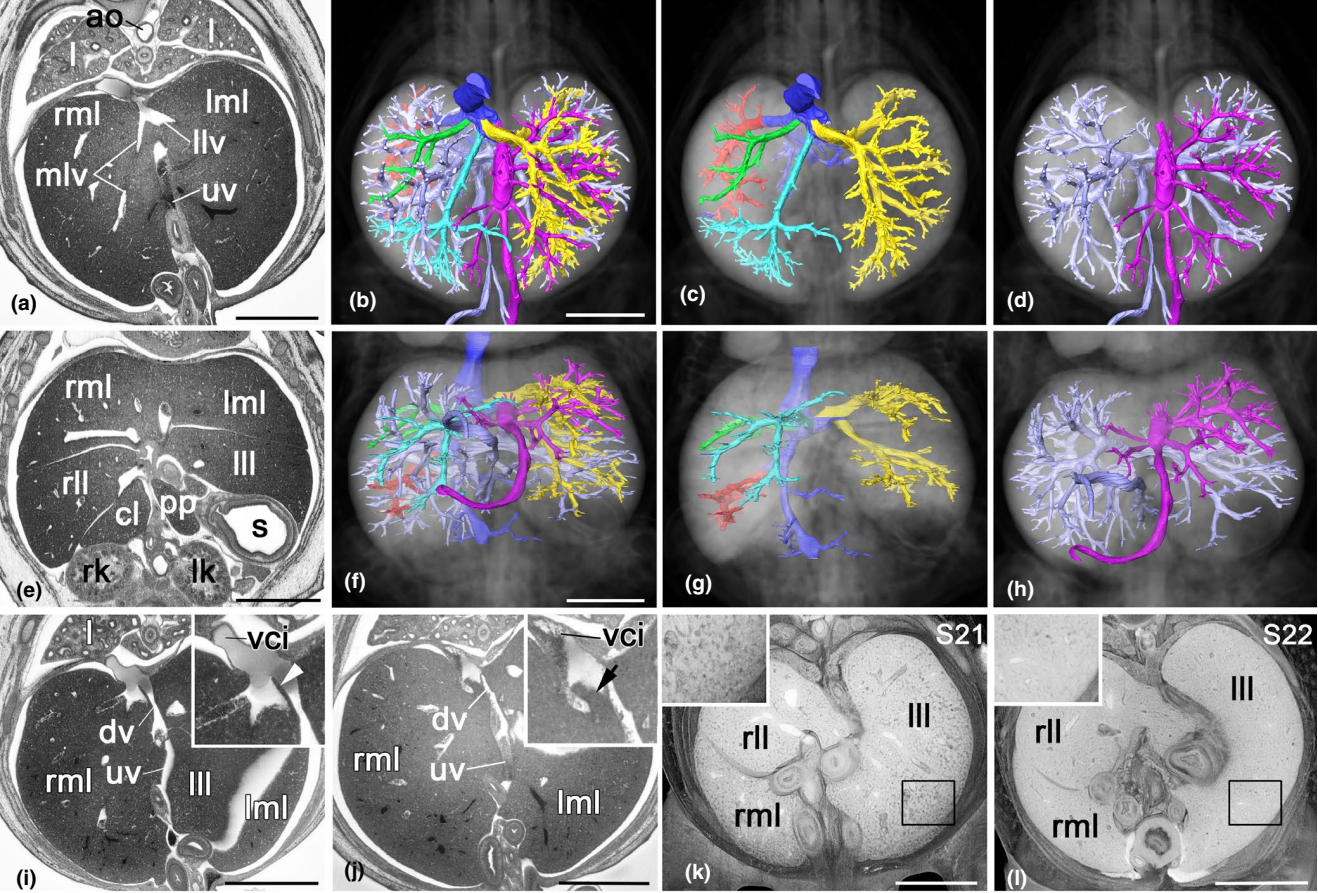
Liver veins, portal venous system and umbilical vein. (a–h) Liver. Axial HREM‐section (a), surface models of veins in context with axially sectioned semi‐transparent volume model from cranial (b, c), coronal HREM re‐section (e), and surface models in context with coronally sectioned semi‐transparent volume models of the liver (f–h). Vena cava inferior (blue), portal venous system (light blue), vitelline vein (light blue), superior mesenteric vein (light blue), umbilical vein and branches (magenta), branches of left liver vein (yellow), middle liver vein (turquoise), right liver vein (green), caudal liver vein (red). Note that the branches of the umbilical vein feed mainly the left medial and parts of the right medial liver lobe. (i, j) Ductus venosus (dv) and valve of ductus venosus. Axial HREM sections. Note the slender appearance of the valve (arrowhead) in (i) and the plump appearance (arrow) in (j). (k, l) Liver texture of a stage 21 (k) and stage (22) embryo (l). Axially sectioned volume model. ao, aorta; cl, caudate liver lobe; c, colon; dv, ductus venosus; j, jejunum; l, lung; lk, left kidney; lll, left lateral liver lobe; llv, left liver vein; lml, left medial liver lobe; mlv, middle liver vein; mv, mesenteric veins; pp, papillary process; rk, right kidney; rll, right lateral liver lobe; rml, right medial liver lobe; s, stomach; uv, umbilical vein; vci, vena cava inferior; vv, vitelline vein. Scale bars 1 mm [Colour figure can be viewed at wileyonlinelibrary.com]

In addition to the ductus venosus, several small and four large veins collecting blood from the liver drain into the inferior vena cava. Three large veins join the segment of the inferior vena cava that runs inside the right medial liver lobe, cranial to its junction with the ductus venosus. These are as follows: (1) a right liver vein, which collects the veins of the posterior part of the right medial liver lobe; (2) a middle liver vein, which collects the veins of the anterior part of the right medial liver lobe and (3) a left liver vein, which collects the veins of the left liver lobes. The left liver vein is a very short vessel that is formed by joining of two large veins, one collecting the veins from the left medial and one collecting those from the left lateral liver lobe (Figure 4).

Caudal to the junction of inferior vena cava and ductus venosus, the fourth large caudal liver vein drains into the inferior vena cava. It collects the blood from the lateral right liver lobe.

Caudal to this connection, several small veins, which drain the quadrate and caudate liver lobes, join separately.

#### 3.2.6 Umbilical vein and ductus venosus

In E14.5 embryos, large segments of the intestine are placed outside the embryo body and inside the physiological umbilical hernia. Several venous channels enter the body through the umbilicus.

The umbilical vein is the dominant vessel. It runs between the layers of the wall of the umbilical hernia and enters the embryo body to the left of the colon. In a single embryo (0.5%), it entered the body to the right of the colon. Inside the body, it heads for the liver and enters the liver tissue at the porta hepatis, between the left and right lateral liver lobes. In 6% of embryos staged as S22+ and older, its diameter narrows at the level of the gall bladder.

Inside the liver, the diameter of the vessel enlarges significantly and it forms a voluminous, elongated and anterior–posteriorly orientated cavity. From this cavity branches emerge, which drain into all parts of the left medial liver lobe and the anteromedial part of the right medial liver lobe (Figure 4). At the posterior aspect of the cavernous part, the left branch of the portal vein joins and a very short ductus venosus starts, which connects to the inferior vena cava. In two‐dimensional axial sections of 98% of the specimens, the connection appears as if secured by a slender valve. In 2%, this structure appears thickened and plump (Figure 4).

#### 3.2.7 Portal venous system

In addition to the umbilical vein, the remnant of a vitelline vein runs between the layers of the umbilical hernia. It enters the embryo body on the right side in all embryos in which gut rotation has not yet taken place (90%). In embryos, in which the rotation of the intestine has started, the entrance position varies according to the entrance position of the large intestine. Hence, in the 12% of the embryos of stage 22+ to 23 in which rotation of the intestine has finished, the vein enters left to the colon. Strikingly, 13% of embryos staged as S21 also show the vein entering on the left side. Inside the embryo body, the vitelline vein heads straight for the liver hilum.

Inside the umbilical hernia, venous channels draining the intestine form the superior mesenteric vein. Running near the superior mesenteric artery, this passes centrally through the umbilicus and heads towards the liver hilum. Near the liver, it forms the portal vein by joining with the vitelline vein and small veins coming from pancreas, spleen and upper intestine. The latter are barely traceable in HREM data routinely produced for DMDD.

The portal vein is of small diameter. It enters the liver and splits into a right and a left branch. The right branch gives rise to vessels supplying the medial and lateral lobes of the right liver and the caudate and papillary lobe. The left branch gives rise to small vessels supplying the left lateral lobe and then joins the much larger umbilical vein (Figure 4).

Running between the layers of the left wall of the umbilical hernia, a small paraumbilical vein regularly enters the embryo body. It has a very small diameter and connects the subcutis of the right abdomen directly to the liver sinusoids by penetrating into the liver tissues right to the porta hepatis.

During E14.5, the volume of the liver increases dramatically. Therefore, the sinusoids appear much larger and the texture of the liver tissue in 2D sections appears differently in the developmental stages of E14.5 (Figure 4). Except for the sinusoids and the ductus venosus, there is no direct connection between the branches of the portal and umbilical vein and the central or hepatic veins.

### 3.3 Venous malformations in embryos with gene deletions

We explored the usefulness of our reference data using them for identifying abnormalities of the venous system in embryos, which miss both alleles of *Atp11a*, *Morc2a*, *1700067K01Rik*, *B9d2*, *Oaz1*, *Celf4 and Coro1c*.

**FIGURE 5:**
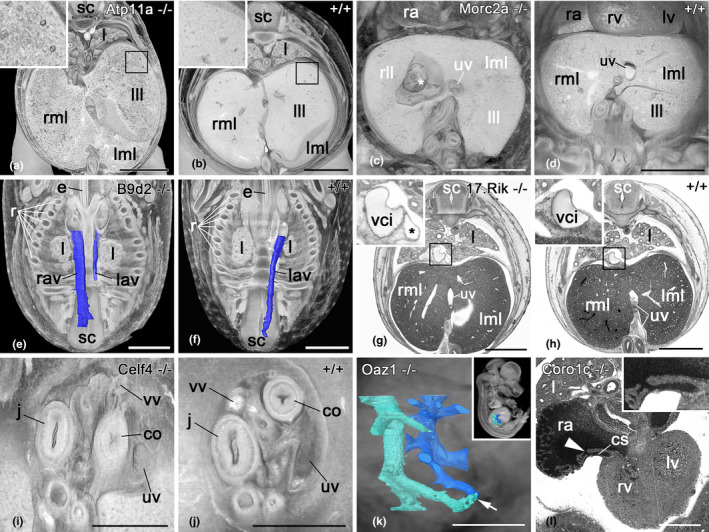
Abnormalities. (a, b) Abnormal liver texture in a *Atp11a* null mutant of stage (S) 22 (a) and stage‐matching control (b). Volume models sectioned axially. (c, d) Liver cyst in a *Morc2a* null mutant (c) and control (d) Volume models sectioned coronally. (e, f) Symmetric azygos vein in a *B9d2* null embryo (e). Control (f). Axial HREM sections. (g, h) Duplication of vena cava inferior in a *1700067K01Rik* null embryo (g). Control (h). Axial HREM sections. (i, j) Abnormal vitelline vein (vv) topology in a S22+ *Celf4* null embryo. Note the position of the vein in respect to the intestine (i). Stage matching control (j). Volume models, sectioned coronally at the basis of the physiologic umbilical hernia. (k) Abnormal liver vasculature in an *Oaz1* null mutant. Note the direct connection (arrow) between branches of the vena hepatica (turquoise) and hepatic portal vein (blue). Surface rendered in context of volume‐rendered model from the right. (l) Abnormal connection of coronary sinus and right atrial appendix (arrowhead) in a *Coro1c* null embryo. Axial HREM section. co, colon; cs, coronary sinus; j, jejunum; l, lung; lav, left azygos vein; lll, left lateral liver lobe; lml, left medial liver lobe; lv, left ventricle; ra, right atrium; rav, right azygos vein; rll, right lateral liver lobe; rml, right medial liver lobe; rv, right ventricle; sc, spinal cord; uv, umbilical vein; vci, vena cava inferior; vv, vitelline vein. Scale bars 1 mm (a–h), 500 µm (i–l) [Colour figure can be viewed at wileyonlinelibrary.com]

Diagnosis of ‘abnormal arrangement of liver veins’, ‘abnormal vitelline vein topology’, ‘abnormal liver texture’ and the additional coronary sinus abnormality all required direct and careful comparisons of the mutants with exactly stage‐matching reference data we created in this study. One *Atp11a* null embryo showed abnormal enlargement of the liver sinusoidal spaces; two *Morc2a* null embryos showed multiple blood‐filled liver cysts, one of them connected to large vasculature; one *1700067K01Rik* null embryo showed a dual inferior vena cava with the additional vein entering directly the left vena cava superior; three *B9d2* null embryo showed an intrathoracic symmetric arrangement of the azygos and accessory azygos veins with the right sided being more voluminous in two of them (Figure 5); one *Oaz1* null embryo showed abnormal arrangement of the liver veins, associated with a very small intrahepatic direct connection between smaller branches of the hepatic portal and hepatic veins; two *Celf4* null embryos showed abnormal vitelline vein topology; and one *Coro1c* null embryo showed an abnormal, dual connection of the coronary sinus. This effectively represented a shunt between the right atrial appendix and the left superior vena cava entering the right atrium.

## 4 DISCUSSION

We present a first, in‐depth, digital volume data‐based description of the venous system of mouse embryos as it presents itself in the E14.5 developmental stages S21–S23 (Geyer et al., 2017b). This is based upon examination of a very large number (206) of wild‐type individuals, bred on a C57BL/6N background as part of the ‘Deciphering the mechanisms of Developmental Disorders (DMDD)’ (www.dmdd.org) program (Mohun et al., 2013). This provided highly detailed 3D information on normal venous anatomy in embryos of each of the six developmental substages of E14.5, yielding a reference framework for diagnosing venous abnormalities in E14.5 embryos of genetically compromised and engineered mouse lines.

High‐resolution episcopic imaging data provide great anatomical detail but space constraints prevent us illustrating the morphology and topology of all the functionally important veins through all the developmental stages of E14.5. However, we are happy to collaborate by providing access to all of our raw data and we are equally happy to provide collaborating scientists with detailed visualisations of veins and situses they are interested in.

Usually, images from mounted histological sections are used as reference for diagnosing venous abnormalities in E14.5 embryos (Kaufman, 1992; Theiler, 1989). Since these are 2D, they do not provide 3D information. This, however, is essential for the correct identification and confirmation of the diagnosis of spatially complex structural abnormalities. Our results are derived from digital volume data produced with the HREM technique. Each HREM dataset was comprised of approximately 3000 inherently aligned digital images of excellent tissue contrasts and details. The voxel dimensions of the 3D volumes were approximately 3 × 3 × 3 µm^3^. Such data provide true 3D information and, as demonstrated by several examples, are sufficient for the detection of abnormalities at all levels of resolution, from the whole organism, down to its tissues.

Except for some notable exceptions, the anatomy of the venous system of the mouse is similar to that of humans. Naturally, there are many differences due to specific organ morphology and topology. The most important larger structural dissimilarities affecting the venous system of mice are as follows: First, a right and left superior vena cava; second, a left‐sided azygos vein; third, a confluence of three lung veins dorsal to the left atrium, from which a single pulmonary vein forms that enters the left atrium; and fourth, a different arrangement of liver veins as a consequence of different liver lobulation (Fiebig et al., 2012). Not surprisingly, our results demonstrate that these differences are already established at E14.5.

But, in addition to these general differences between mice and man, there are also differences between adult and unborn mice. The most important is the existence of an umbilical vein and a ductus venosus in the unborn. As a consequence of their presence, the liver vasculature of E14.5 embryos and adult mice differ dramatically. In late foetuses, newborns and adults, venous blood is primarily fed to the sinusoids of all liver lobes via branches of the hepatic portal vein. In E14.5 embryos, the sinusoids of the left medial liver lobe and the anteromedial part of the right medial liver lobe receive blood directly from branches of the umbilical vein. Therefore, these parts of the liver in E14.5 embryos are supplied with blood of high oxygenation and rich in nutrients, whereas the rest receives poorly oxygenated blood from the intestine. The surface‐rendered HREM‐based models we provide permitted the precise visualisation of the blood vessel pattern and a precise definition of the liver volumes concerned.

E14.5 is the transition from the embryonic to foetal period. Hence, many vascular remodelling processes have not yet started or are about to start. Therefore, there are differences in the formation of the hepatic portal vein, the topology of the left renal vein and the appearance of the forming head veins and sinus durae matris.

The hepatic portal vein of E14.5 embryos appears as if formed by the confluence of the superior mesenteric and a vitelline vein, rather than by a confluence of superior mesenteric and splenic veins. In contrast to adults, the latter are very small and barely visible in routine DMDD HREM data. The left renal vein on the other hand is formed as in adults, but takes a different course. While crossing the aorta, it is entirely and thickly surround by material of the paraaortic bodies.

Most obviously, the venous system in the head region is heavily remodelled between E14.5 and adult life. E14.5 embryos do still have a primary head sinus (Padget, 1956) as the main drainage. As our HREM study clearly visualises, this vein is gradually replaced by the forming sigmoid sinus during E14.5. In addition, there are other sinuses, which have not yet come into existence. The superior ophthalmic vein, which starts as a large vessel in the orbit, continues directly into the primary head sinus at S22 and earlier. At stage 22+ and later, it appears to drain into the junction of transverse and sigmoid sinus, passing basically without notable morphological alteration through the very locations where the cavernous and superior petrosal sinus form. This is rather surprising and suggests that the venous plexus of the parasellar region (Weninger & Müller, 1999; Weninger & Pramhas, 2000) does not form around an existing vein, but connects to it in later developmental stages. This is only hypothetical and has to be verified in future studies. An interesting finding is also that the dimensions of the primitive left and right transverse sinus differ markedly, with the left usually being larger than the right. This asymmetry gradually decreases with maturation of the sinus system. Future studies, including earlier developmental stages are required to research the cause of such changes.

Remodelling of the sinus system, with the sigmoid sinus forming, the primary head sinus vanishing and the transverse sinus becoming symmetric, provides the clearest example of why an accurate staging is a prerequisite for the correct interpretation of the phenotype of E14.5 mice. However, it is also essential to be aware of the various stage‐specific and unspecific venous peculiarities in S21 to S23 embryos. These include the architecture of the liver sinusoids and liver vasculature, the connection of vitelline vein topology and physiological gut rotation (Geyer et al., 2017b), the occasional narrowing of the umbilical vein and even the connection of the coronary sinus to the right atrium.

Only by relying on precise descriptive reference data can pathologies such as abnormal liver tissue composition, abnormal ductus venosus valve morphology, intrahepatic shunts between hepatic portal and hepatic venous system be securely defined as malformations. Our study has, for example, identified dual connection of the coronary sinus, a pathology affecting some embryos which was previously unknown. Establishing that a double connection was not a normal but transitory feature at E14.5 required detailed reference data derived from significant numbers of normal embryos at each of the successive stages of E14.5. It remains to be examined in future studies whether this double connection affects haemodynamics is a source for thromboembolic brain ischemia or has any other pathologic relevance. Finally, it has to be emphasised that precise descriptions of the stage dependency of anatomical features not only add to our knowledge of normal development. It is also the basis for the accurate detection of heterochronic events, since simple comparisons of mutants with single—possibly not stage matching—controls might lead to misdiagnoses (Boughner et al., 2018; Geyer et al., 2017b).

The DMDD programme showed that the penetrance of phenotypes varies substantially between different embryos (Wilson et al., 2016a). Heavily affected individuals of a single knockout line with, for example, severe cardiovascular malformations might die before E14.5. Less severely affected embryos might survive. If characteristic mild venous abnormalities can be linked with more severe and lethal cardiovascular defects, this might provide a way to use the mild abnormalities as indicators of those that are more severe. Presence of the ‘indicator’ in embryos surviving until E14.5 might indicate that some littermates with low penetrating, but severe malformations, have already died. In such cases, the presence of the indicator in E14.5 mice would suggest that larger numbers including younger individuals of a knockout line should be examined to reveal the full phenotype spectrum arising from the mutation. Such ‘indicators’ have already shown to exist for central nervous system malformations (Reissig et al., 2021). Whether and which venous variations, features and abnormalities might have a potential to serve as indicators for severe malformations of the cardiovascular or other organ systems remains to be explored.

Our data are based on embryos of the C57BL/6 strain, which is one of the most popular mouse strains of modern biomedicine and had been selected by the IMPC and DMDD as its standard genetic background strain. Thus, the reference data we have presented here may be useful for the broader scientific community working with this mouse strain. To what extent the current data can be transferred to other popular mouse strains remains to be assessed. While there is only scarce information on head veins, some strain peculiarities have been documented that affect the azygos vein and liver anatomy (Biddle et al., 1991; Fiebig et al., 2012).

The results from the DMDD phenotype screen were made publicly available via the DMDD website (www.dmdd.org.uk) as well as the mouse genome informatics site of the Jackson Laboratory (http://www.informatics.jax.org/). This also applies for the knockout embryos we included in this study. While we merely concentrate on selected venous abnormalities, the full phenotype and an estimation of the penetrance of the single features can be deduced from visiting these pages and some of the lines have already started to be subject of more systematic study (e.g. De Franco et al., 2019; Perez‐Garcia et al., 2018; Reissig et al., 2019).

## CONFLICT OF INTEREST

No conflict of interest declared.

## AUTHORS’ CONTRIBUTIONS

SHG and WJW designed the study, drafted and revised the manuscript. FP, RW, AG, CT, JKW and TJM created mouse lines and HREM data. LFR and WJW did phenotyping. SHG, BMG and JR created 3D models and did statistics.

## Data Availability

The data that support the findings of this study are available from the corresponding author upon reasonable request.
